# Comparison of Laparoscopic and Conventional Cystotomy/Partial Cystectomy in Treatment of Liver Hydatidosis

**DOI:** 10.1155/2019/1212404

**Published:** 2019-02-05

**Authors:** Huseyin Kazim Bektasoglu, Mustafa Hasbahceci, Yunus Tasci, Ibrahim Aydogdu, Fatma Umit Malya, Enver Kunduz, Kemal Dolay

**Affiliations:** ^1^Department of General Surgery, Faculty of Medicine, Bezmialem Vakif University, Vatan Street, Fatih, 34093 Istanbul, Turkey; ^2^Department of Pediatric Surgery, Faculty of Medicine, Bezmialem Vakif University, Vatan Street, Fatih, 34093 Istanbul, Turkey; ^3^Department of General Surgery, Liv Hospital Ulus, Istinye University, Besiktas, 34340 Istanbul, Turkey

## Abstract

**Introduction:**

Hydatidosis is a zoonotic infection and treatment is mandatory to avoid complications. Surgery remains the first choice in the treatment especially for CE2-CE3b cysts. Open or laparoscopic approaches are available. However, comparative studies are limited.

**Materials and Methods:**

Data of patients who underwent cystotomy/partial cystectomy for liver hydatidosis between January 2012 and September 2016 (n=77) were evaluated retrospectively. Recurrent cases and the patients with previous hepatobiliary surgery were excluded. 23 patients were operated upon laparoscopically and named as Group 1. 48 patients operated conventionally named as Group 2. Demographics, cyst characteristics, operative time, length of hospital stay, recurrences, and surgery related complications were evaluated.

**Results:**

Groups were similar in terms of demographics, cyst characteristics, and operative time. The length of hospital stay was 3.4 days in Group 1 and 4.7 days in Group 2 (p=0,007). The mean follow-up period was 17.8 months and 21.7 months, respectively (p=0.170). Overall complication rates were similar in two groups (p=0.764). Three conversion cases occurred (13%). One mortality was seen in Group 2. Four recurrences occurred in each group (17% versus 8.3%, respectively) (p=0.258).

**Conclusions:**

Laparoscopy is a safe and feasible approach for surgical treatment of liver hydatidosis. Recurrence may be prevented by selection of appropriate cases in which exposure of cysts does not pose an intraoperative difficulty.

## 1. Introduction

Hydatidosis is a zoonotic infection caused by a parasite Echinococcus granulosus and endemic mainly in the Mediterranean area, the Middle East, China, and the Far East [1, 2]. Although the liver is the most commonly affected organ (50-70%), the spleen, lungs, the kidney, muscles, bones, and the brain may be affected less frequently [3–5]. Most patients are asymptomatic and the diagnosis is made incidentally. However, presenting symptoms may include abdominal pain, palpable mass, jaundice, fever, and anaphylaxis. Treatment of viable hepatic hydatid cysts is mandatory due to risk of complications such as rupturing into peritoneum, cholangitis, portal hypertension, and secondary hydatidosis [6–8].

Treatment options are medical therapy, percutaneous interventions, and surgery. Surgery remains the gold standard in the treatment of hydatid cyst especially in CE2-CE3b cysts [9, 10]. Surgical operations may vary from radical procedures such as total pericystectomy and hepatectomy to more conservative procedures such as cystotomy plus drainage and partial cystectomy with or without omentopexy. These procedures may be performed through either open or laparoscopic approach. Open surgical procedures may cause incision related complications (i.e., surgical site infections, abdominal hernias, and increased pain), increased scar formation, longer hospital stay, and recovery [11].

Laparoscopic treatment of hepatic hydatidosis was first reported in 1992 and became popular in recent years [12]. Minimal invasive approach has advantages of reduced incisional complications, faster surgery, shorter hospital stay, less postoperative pain, and good cosmetic results [9, 11, 13–15]. However, it is not free from several disadvantages including potential risk of spillage, required learning curve, potential bleeding risk, and longer operation times [10, 16, 17].

Comparative studies of laparoscopic and open approaches in the treatment of liver hydatidosis have been limited in literature. Controversies about the role of laparoscopy such as patient selection and the differences in surgical techniques have not yet been resolved. This study was aimed at comparing the results of laparoscopic and conventional open approaches to liver hydatidosis.

## 2. Materials and Methods

After obtaining institutional board review approval, data of patients who underwent cystotomy/partial cystectomy for liver hydatidosis between January 2012 and September 2016 (n=77) were evaluated retrospectively. Diagnosis was made by conventional imaging (ultrasonography, computed tomography, or magnetic resonance imaging) and serology. Recurrent cases (n=4) and the patients with previous hepatobiliary surgery (n=2) were excluded. Therefore, a total of 71 patients were included in this study. All patients were treated with albendazole (10 mg/kg, Andazol, Biofarma Drugs, Istanbul, Turkey) two to three weeks prior to the operation and three to six months following the operation. Appropriate antibiotic prophylaxis was performed 30 minutes prior to the operation for all patients. 23 patients who were operated upon via laparoscopic approach were named as Group 1. 48 patients who were operated upon via conventional open approach were named as Group 2. A total of five different surgeons performed the operations. Three of them were specialized in hepatopancreatobiliary surgery and the remaining two were practicing as general surgeons. Demographic data (age, gender), imaging characteristics (location, number, maximum diameter, and concomitant biliary connection) of hydatid cysts, length of hospital stay, recurrences and surgery related complications (purulent drainage, biliary drainage, requirement of endoscopic intervention, superficial surgical site infection), and mortality were evaluated. Primary end point of this study was defined as the development of surgery related complications.

### 2.1. Statistical Analysis

Data were analyzed via using SPSS v20 software. Categorical variables were evaluated with Pearson chi square test. Other quantitative parameters were evaluated with Mann-Whitney* U* test. P values lower than 0.05 were considered as statistically significant.

## 3. Surgical Technique

### 3.1. Laparoscopy

**Figure 1:**
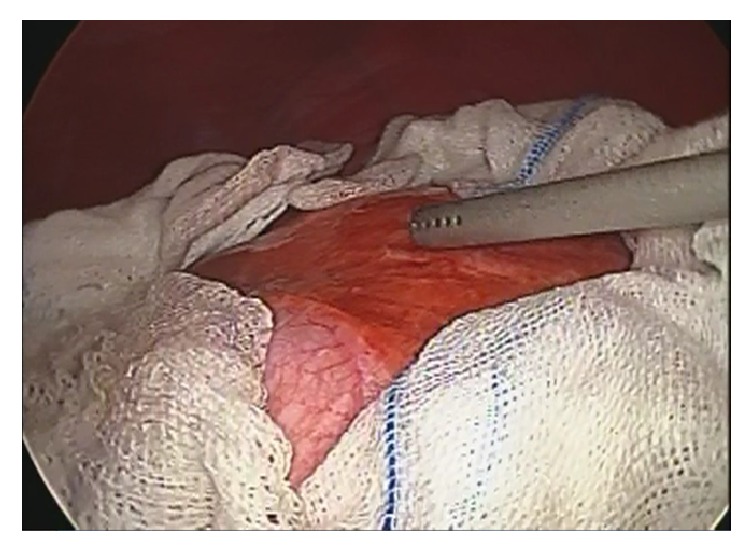
Laparoscopic surgical field with gauzes location.

Four ports were inserted: supraumbilical 10 mm port with 30 degree telescope, 10 mm epigastric port, and additional two ports that were depending mainly on the cyst location for each patient. Pneumoperitoneum were set at 12 mmHg. Gauzes soaked with 20% hypertonic saline were placed around the cysts (Figure 1). The cyst was punctured and aspirated with 10 mm laparoscopic aspirator. The 20% hypertonic saline was used as scolicidal agent and injected into the cyst cavity. After 10 minutes, the cyst was aspirated again. Cystotomy was performed via electrocautery and the cavity was carefully explored via telescope for biliary leakage from the inner side of the cyst wall. If biliary leakage was detected, laparoscopic sutures or clips were used to ligate the biliary fistulous connection. The cyst cavity was irrigated with 20% hypertonic saline several times. Partial cystectomy was performed according to superficial localization of cyst. A rubber suction drain was placed into the cyst cavity. Oral fluid intake was allowed at the postoperative 6^th^ hours. Drain was removed after 48 hours if there is no apparent bile. The patients were in follow-up period scheduled to three to six-month interval with imaging, liver function tests, and serology if necessary.

### 3.2. Open Approach

A right subcostal or upper median incision was preferred due to cyst localization. Surgical technique was similar to laparoscopic surgery. 20% hypertonic saline was used as scolicidal agent. An open aspirator and surgical spoon were used for suction and clearance of the cyst cavity. Postoperative follow-up procedure was similar to in the laparoscopic group.

## 4. Results

There were 23 patients with mean age of 39.4 ± 19.1 years in Group 1. Male to female ratio was 11/12. Group 2 included 48 patients with a mean age of 41 ± 15.4 years. Male to female ratio was 25/23. There was no statistically significant difference between two groups in terms of gender and age (p=0.740 and p=0.631, respectively). Cyst characteristics were presented in Table 1. In addition, four cases (17.3%) in Group 1 and eight cases (16.6%) in Group 2 had cysts located in segment 7 according to Couinaud classification (p=0.939).

The mean operative time was 150 ± 63 minutes in Group 1 and 113 ± 63 minutes in Group 2 (p=0,013).

The length of hospital stay was 3.4 ± 1.4 days in Group 1 and 4.7 ± 2.2 days in Group 2. Length of hospital stay was significantly longer in Group 2 (p=0.007).

The duration of follow-up period was 17.8 ± 9.3 months and 21.7 ± 11.1 months in Groups 1 and 2, respectively (p=0.170). In this period, seven patients (30%) in Group 1 and 13 patients (27%) in Group 2 suffered from complications (p=0.764). Details of the complications were presented in Table 2.

In group 1, laparoscopic hemoclip was used in one patient and suture ligation was used in three patients to control the suspected bile leak. In Group 2, suture ligation was performed in ten patients to control the bile leak. Biliary leakage following surgery was detected 13% (3/23) and 15% (7/48) in the laparoscopy (Group 1) and open surgery (Group 2) groups, respectively (p=0.861). However, persistent bile fistula was seen in one patient in Group 1 and four in Group 2; all patients underwent endoscopic interventions, and all resolved.

Although there was no mortality in Group 1, one mortality was seen in Group 2 due to postoperative hepatic encephalopathy. Four recurrences were seen in Group 1 (17%) and Group 2 (8.3%) (p=0.258).

## 5. Discussion

Hydatid disease is a major health problem in endemic areas. The liver is commonly affected organ that accounts 70% of the cases and most commonly the right lobe is implicated [3–5, 10]. Treatment options include medical therapy, percutaneous interventions, and surgery. Among all treatment modalities, surgery remains the mainstay of the treatment especially in CE2-CE3b cysts [9, 10].

Surgical strategies may vary from conservative methods such as cystotomy plus drainage and partial cystectomy to more radical methods such as lobectomy and hepatectomy. Although open procedures have usually been performed for the surgical treatment of hydatid disease, laparoscopic approaches have become popular in recent years.

Laparoscopic surgery for the hydatid disease obtains the advantages of minimal invasiveness such as lesser incisional complications, faster surgery, shortened hospital stay, and good cosmetic results [9, 11, 13–15]. On the other side, risk of intraoperative spillage, requirement of learning curve, and risk of intraoperative bleeding have been claimed to be disadvantages of laparoscopic approach to liver hydatidosis [1, 10, 16, 17]. Therefore, laparoscopic approach can be selected based on the surgeon's preference and it can be expected to be the main approach for the surgical treatment of liver hydatidosis during the next years despite its possible disadvantages.

Conversion rates in laparoscopic surgery for liver hydatidosis have been reported as 4-30% in previous reports [7, 18–20]. Inappropriate exposure of cysts, intra-abdominal adhesions, and bleeding has been reported as the most common causes [7, 18–20]. In the present study, conversion rate was detected as 13% (3 out of 23). Intra-abdominal adhesions and intraoperative anaphylaxis were the causes for conversion. The anaphylaxis occurred while laparoscopic manipulation of the cyst wad before evacuation of the ingredients. As a consequence, the surgeons dealing with laparoscopic treatment of liver hydatidosis should keep in mind that conversion may be necessitated as a consequence of several factors more commonly in comparison to laparoscopic approach for other diseases.

Another intraoperative complication of liver hydatidosis is the development of biliary fistulous tract between the cyst cavity and the biliary system that has been reported as 3-17% in the literature [21]. Therefore, detection of bile staining in the cyst cavity and cessation of bile leakage has a prominent importance to avoid increased risk of postoperative complications. Tuxun et al. [10] reported postoperative bile leakage as 6.24% among 914 patients with liver hydatidosis treated via laparoscopically. A careful exploration of cyst cavity via optic camera as an advantage of laparoscopy may help physicians to detect biliary leakage. Although we could not evaluate the possible association between laparoscopic exploration of the cyst cavity and the detection rate of biliary leakage, it may be recommended to do several attempts to explore cyst cavity.

Length of hospital stay may vary according to the preferred surgical modality. Shortened hospital stay has been reported in laparoscopic liver hydatidosis surgery when compared to open techniques in the literature [9, 11, 14, 22]. In a retrospective analysis of 83 patients in which 14 of them treated laparoscopically, Bostanci et al. [23] reported the mean length of hospital stay was 5.4 day shorter in laparoscopic group (3.4 versus 8.8 days). Ertem et al. [19] reported the length of hospital stay as 4.2 days for 48 laparoscopically treated patients. In this study, as compatible with the previous reports, the length of hospital stay has been found to be significantly shorter in the laparoscopy group than that of the open group (3.4 versus 4.7 days). Therefore, length of hospital stay favors laparoscopic approach for the surgical treatment of liver hydatidosis.

Mortality rates have been reported as 0-6.5% for hydatid disease surgery [24, 25]. In this study, no mortality was seen in the laparoscopy group. However, one mortality case occurred in the open surgery group due to postoperative hepatic encephalopathy in which the patient had been suffering from liver cirrhosis.

Recurrence is one of the major problems in liver hydatidosis surgery and has been reported around 10% in the literature [26]. Common causes of the recurrences have been reported as remnant daughter vesicles and intraoperative spillage [9, 27]. Therefore, some authors suggest that open approach should be performed for posteriorly located cysts due to difficulty of intraoperative exposure [10, 28]. Recurrence rate in the open surgery group was 8.3% (4/48) and it was regarded as compatible with the previous reports. However, the laparoscopic group had 17% (4/23) recurrence rate. Khoury et al. [29] reported three recurrences among 83 laparoscopically treated patients and Seven et al. [30] reported one recurrence among 33 patients. In this study, this high recurrence rate may be conducted with the cyst localization. In two of four recurrent cases in the laparoscopy group, the hydatid cysts were located at segment 7 and difficult intraoperative exposure might cause the retained daughter cysts within the cavity. Although there was no intraoperative spillage as a cause for recurrence, wide spread use of laparoscopy might be another factor for recurrence due to the insufficient clearance of the cyst cavity. Therefore, future prospective studies are required to clarify possible risk factors for recurrence after laparoscopic hydatid cyst surgery.

The operative time was longer in Group 1, and the difference reached a statistically significant level (p=0,013). We think that the operative time may be affected from heterogeneity of surgical team and would be decreased with the increased experience.

Main limitations of this study were retrospective design, relatively small number of the cases, relatively short follow-up time, and unfeasible design to evaluate the learning curve of laparoscopy due to the several surgeons took participate in these operations.

## 6. Conclusion

Laparoscopy is a safe and feasible approach for the surgical treatment of liver hydatidosis. Recurrence may be prevented by selection of appropriate cases in which exposure of cysts does not pose an intraoperative difficulty.

## Figures and Tables

**Table 1:** Details of cyst characteristics according to groups.

	Group 1	Group 2	p
	(n=23)	(n=48)	
Maximum diameter (mm)	95.4 ± 39.6	106.7 ± 42.5	0.273

Cyst localization			0.336
(i) Right lobe	14	32	
(ii) Left lobe	7	8	
(iii) Both lobes	2	8	

**Table 2:** Details of the postoperative complications.

	Group 1 (23 patients)	Group 2 (48 patients)
Purulent drainage	1	2

Biliary drainage	3	7

Postoperative endoscopic intervention requirement	1	4

Superficial surgical site infection	1	7

Anaphylaxis	1	0

Conversion to open surgery	3	NA*∗*

*∗* Note that ten complications in seven patients were seen in Group 1 and 20 complications in 13 patients were seen in Group 2. NA: not applicable.

## Data Availability

The data used to support the findings of this study are available from the corresponding author upon request.
